# Identification of Serum Ferritin-Specific Nanobodies and Development towards a Diagnostic Immunoassay

**DOI:** 10.3390/biom12081080

**Published:** 2022-08-05

**Authors:** Yaozhong Hu, Jing Lin, Yi Wang, Sihao Wu, Jing Wu, Huan Lv, Xuemeng Ji, Serge Muyldermans, Yan Zhang, Shuo Wang

**Affiliations:** 1 Research Institute of Public Health, School of Medicine, Nankai University, Tianjin 300071, China; 2 Laboratory of Cellular and Molecular Immunology, Vrije Universiteit Brussel, 1050 Brussels, Belgium

**Keywords:** serum ferritin, nanobody, diagnosis, immunoassay, clinical indicator

## Abstract

Serum ferritin (SF) is an iron-rich protein tightly connected with iron homeostasis, and the variations are frequently observed in diseased states, including iron-deficiency anemia, inflammation, liver disease, and tumors, which renders SF level an indicator of potential malignancies in clinical practice. Nanobodies (Nbs) have been widely explored and developed into theranostic reagents. Surprisingly, no reports stated the identification of anti-SF Nbs, nor the potential of such Nbs as a diagnostic tool. In this study, we generated SF-specific Nbs and provided novel clinical diagnostic approaches to develop an immunoassay. An immune library was constructed after immunizing an alpaca with SF, and five Nbs specifically targeting human SF were retrieved. The obtained Nbs exhibited robust properties including high stability, affinity, and specificity. Then, an ELISA-based test using a heterologous Nb-pair was developed. The calibration curve demonstrated a linear range of SF between 9.0 to 1100 ng/mL, and a limit of detection (LOD) of 1.01 ng/mL. The detecting recovery and coefficient variation (CV) were determined by spiking different concentrations of SF into the serum sample, to verify the successful application of our selected Nbs for SF monitoring. In general, this study generated SF-specific Nbs and demonstrated their potential as diagnostic immunoassay tools.

## 1. Introduction

Ferritin is an iron-storage protein, critically involved in iron homeostasis or metabolism through either intracellular or secreted extracellular proteins. Ferritin is a heterogeneous polymer of 24 subunits classified as heavy (H) or light (L) type units to form a roughly spherical cage with an outer shell of apoferritin and the internalized ferric iron within inner cores [1,2,3]. The ratio of H and L subunits in ferritin varies according to its origin or the internal healthy or dysfunctional microenvironment during inflammation or infection. The secreted ferritin in the vascular system is referred to as serum ferritin (SF), which is mainly composed of L-type subunits and relatively poor ferric iron [4]. The SF is frequently employed as the clinical indicator of internal iron level to support the prognosis of an iron deficiency or overload, which are tightly connected to the morbid state of chronic and acute diseases. Generally, normal SF levels range from 30–300 ng/mL and 15–200 ng/mL for men and women, respectively [5,6]. Numerous studies have shown that the increase or decrease of SF is closely related to diseases such as iron-deficiency anemia, inflammation, tumors, and cancer, etc. Therefore, SF monitoring has been used as an important indicator for the pre-indication and diagnosis of potential diseases, such as a supportive tool in the diagnosis of clinical anemia, especially iron deficiency (ID) anemia [1,7]. It is estimated that around one-third of the world’s population suffers from anemia with an extremely significant impact on women, especially during pregnancy which can cause maternal or fetal morbidity and mortality [8,9,10]. In addition, evidence has accumulated over time, linking an increased SF level with various malignancies, including inflammatory and tumoral diseases, such as cirrhosis, hepatoma, and other solid tumors [11,12,13,14]. The determination of SF levels can facilitate the prognosis of internal homeostasis and can be used as a relevant basis for the initial diagnosis of several diseases with an underlying ID anemia complication [15,16]. ID during the second and third trimesters of pregnancy increases the risk of anemia after birth. From this point of view, it is essential to monitor the specific situation of maternal iron reserves during the corresponding period of pregnancy [17]. An SF level below the lowest of the normal range is considered the most relevant biochemical indicator of ID, which has been verified by multiple studies to qualify as being the most representative test for ID screening during pregnancy [18,19]. Therefore, it is crucial to use an appropriate assay to measure serum ferritin levels to indicate the symptom of ID, as well as the connection of homeostasis with potential diseases [20].

Currently, detection methods such as radioimmunoassay (RIA), electro-chemiluminescence assay (ECLIA), and enzyme-linked immunosorbent assay (ELISA) have been developed to determine the SF content [21,22]. All of these detection techniques depend on the preparation of specific conventional monoclonal antibodies (mAbs) to form antibody–SF complexes and provide efficient identification of SF with adequate specificity and sensitivity [23,24]. All methods listed above are applied in the clinical diagnosis of SF, whereas RIA and ECLIA are the preferred methods due to their high sensitivity and repeatability. Conversely, the main disadvantages of these technologies include the possible radioactive contamination, the short effective storage time of reagents, and high cost. Hence, ELISA remains an attractive alternative and useful method to monitor the SF level at a low cost and with convenient operation. However, the intrinsic drawbacks and limitations of mAbs, (i.e., a labor-intensive selection process and targeting surface epitopes that frequently cross-react with other undefined epitopes and their tedious manipulation) have restricted the further development of mAb-based detecting methods. Here, we propose to substitute the mAbs with Nanobodies as a novel antibody candidate for SF detection. We evaluated their biochemical properties with the aim of applying these Nanobodies as detecting reagents, and most importantly to demonstrate their applicability for SF surveillance. The nanobody (Nb, also known as VHH) as an alternative antibody format is the single domain antigen-binding fragment derived from the heavy chain-only antibody (HCAb) naturally occurring in camelids [25,26]. The straightforward selection via phage display of specific Nbs against virtually any target of interest has been established and retrieves effective affinity reagents for diagnosis and therapy [27,28]. More importantly, the unique paratopes of Nbs facilitate the targeting of their cognate antigens with high specificity and affinity and even allow the binding to the epitopes that are less accessible to conventional antibodies [29,30]. The excellent biochemical characteristics of Nbs, including the good solubility in aqueous solvents and high stability even under harsh conditions such as high temperature, extreme pH, and presence of chemical denaturants, qualified Nbs as promising candidates to develop diagnostic reagents for clinical applications [31,32].

In this study, we developed Nbs with specificity against a clinical diagnostic indicator of SF and evaluated their potential as a tool for SF surveillance. In general, an immune Nb library was constructed after consecutive immunization with the antigen protein, which served as the Nb repertoire to retrieve SF-specific Nbs after several rounds of bio-panning and screening. The characteristics, including specificity, affinity, and thermal stability of the retrieved Nbs have been measured to identify the lead Nbs. The targeting of the selected Nbs to SF has been verified by immunoprecipitation or blotting assay. The potential of selected SF-specific Nbs as diagnostic reagents has been confirmed by developing a heterologous Nb-pair-based sandwich ELISA with sufficiently high sensitivity and good reproducibility.

## 2. Materials and Methods

### 2.1. Protein, Strains, and Plasmids

The antigen protein of SF used for animal immunization was purchased from Kitgen Bio-tech, China. *E. coli* TG1 competent cells were used for the construction of the immune library and purchased from Lucigen, Middleton, WI, USA. *E. coli* WK6 cells from our lab strain collection were used for Nb expression. The pMECS phagemids were used for the preparation of the Nb-displayed phage reservoir, and the expression of Nbs fused with HA- and His-tag. The plasmid of pHEN6c was used for the expression of Nbs fused with only His-tag [33]. All the plasmids were available from our laboratory collection.

### 2.2. Immunization and Immune Nb Library Construction

**Figure 1:**
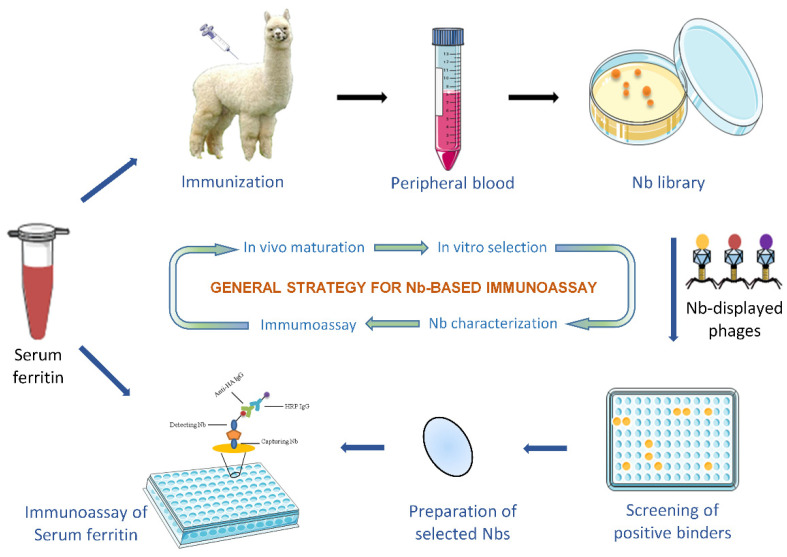
Schematic illustration of SF-specific Nb preparation and development of an immunoassay.

Immunization occurred via subcutaneous injection of SF into a young alpaca according to the well-established protocol with minor modifications [34]. Animal experiments were performed in accordance with the guidelines of the Animal Care and Use Committee of Nankai University (2022-SYDWLL-000026). The general strategy is schematically illustrated in Figure 1. Generally, 0.1 mg of thoroughly emulsified SF was injected to raise an immune response during the 6-week duration of the immunization with one injection per week. Three days after the last injection, 50 mL of anti-coagulated blood was drawn and subjected to the next step to isolate peripheral blood lymphocytes by using Lymphoprep™ Density Gradient Medium (STEMCELL Technologies, Vancouver, BC, Canada). The total RNA was extracted from the obtained lymphocytes by using TRIzol™ Reagent (Invitrogen, Carlsbad, CA, USA) to facilitate the preparation of first-strand cDNA. Then, the genes encoding VHH fragments were amplified by performing two rounds of nested PCR. In the first round of PCR, primers CALL001 and CALL002 (Table S1) were employed to generate amplicons corresponding to the variable and constant domains (VH-CH1-CH2 around 900 bp; VHH-CH2 around 700 bp). The fragments of around 700 bp were extracted by QIAquick Gel Extraction Kit (QIAGEN, Hilden, Germany) after separation by agarose gel electrophoresis to serve as the template in the second PCR to facilitate the preparation of VHH fragments by using the primers of PMCF and A6E (Table S1) to introduce Not I and Pst I restriction enzyme sites, which contribute the insertion of the VHH amplicons into the pMECS phagemid vector after digestion with corresponding restriction enzymes (NEB, Ipswich, MA, USA). The resulted recombinant phagemids containing VHH repertoire were then electro-transformed into *E. coli* TG1 competent cells. The size of the library was calculated by counting the number of colonies (transformants) after serial dilutions plated on Luria–Bertani (LB) agar plates. The remaining TG1 transformants were then plated on large Petri dishes, to constitute the immune library. The colonies (confluent) formed after overnight incubation at 37 °C were scraped from the plates to obtain the immune Nb library. The insertion of a PCR amplicon with the size of a VHH in the pMECS vector was determined by colony PCR with primers GIII and MP57 (Table S1) on randomly picked colonies.

### 2.3. Bio-Panning and Screening of Specific Nbs

Enrichment of phages displaying SF-specific Nbs was performed by consecutive rounds of bio-panning based on the well-described protocol [35]. Briefly, a representative aliquot of the immune Nb library was inoculated into 300 mL of 2◊TY medium and shaken until the exponential growth phase was reached. M13KO7 helper phages were added to rescue the pMECS phagemid into virions with surface displayed Nbs. After overnight incubation, phage particles with displayed Nbs in the culture supernatant were collected after centrifugation and precipitated by adding polyethylene glycol (PEG)/NaCl (1:10 (*v*:*v*)) and 1 h incubation on ice. PEG-insoluble phages were then pelleted by centrifugation and resuspended in 1 mL of sterile PBS. Wells of microtiter plates pre-coated with 100 μL of SF (1 μg/mL) were used to enrich virions carrying target-specific Nbs, and wells without SF coating served as negative control (background enrichment). After blocking with 200 μL of 3% (*w*/*v*) skimmed milk, 1 × 10^11^ PFU of phages were added into the wells coated or non-coated with SF, to allow the binding of phage-displayed Nbs to antigen. The stringent washing steps with PBST (PBS containing 0.05% Tween-20) were applied to remove unbounded phages, and the remaining phages were eluted by incubating with 100 μL TEA solution (100 mM triethylamine, pH 11.0) for 10 min at RT. Then, the collected phage particles were neutralized in 100 μL of Tris-HCl (1.0 M, pH 7.4). The relative enrichment was determined by counting the colonies from serial dilutions of SF-positive wells and SF-negative wells after infecting fresh TG1 cells with 10 μL of corresponding phage particles. The remaining collected phage particles were used to infect TG1 cells for the amplification and preparation of phages, taken to the next round of bio-panning.

The screening of SF-positive binders was initiated when a relatively high enrichment was obtained during bio-panning. Single colonies were randomly picked from the plates with enriched colonies and individually inoculated into 100 μL 2× TY medium in 96-well culture plates. After overnight incubation at 37 °C without shaking, 10 μL of cell culture were added into the corresponding wells of a 96-deep well plate and incubated at 37 °C with shaking until the exponential phase was reached. Then, isopropyl β-D-1-thiogalactopyranoside (IPTG) was added to induce the production of Nbs. Cells were collected by centrifugation on the next day to allow the release of periplasmic extracts (PE) through freeze-thaw cycles and then applied to the wells with or without prior SF coating, for the selection of positive colonies in a PE-ELISA. The wells containing potential positive SF binders were determined according to the reading signal at 405 nm after sequential incubation with mouse anti-HA IgG (1:5000) (Invitrogen, Waltham, MA, USA), alkaline phosphatase (AP) conjugated goat anti-mouse IgGs (1:5000) (Invitrogen), and substrate for color development. The wells with a 2-fold higher signal compared to the wells without SF were considered as containing extracts from clones producing potential binders of SF and were selected for large-scale plasmid preparation and nucleotide sequence analysis.

### 2.4. Expression and Purification of Specific Nbs

After the selection of SF-binders, the recombinant pMECS plasmids containing the encoding genes of Nbs were transformed into *E. coli* WK6 cells. *E. coli* TG1 cells suppress the amber stop codon between VHH and the gene III in pMECS phagemid, resulting in the expression of VHH-pIII fusions. However, *E. coli* WK6 is a non-suppressor strain, and will not yield fusion Nbs with pIII, but only Nbs with HA- and His-tag at their C-terminal end. Moreover, soluble Nbs can be extracted from the periplasm by osmotic shock for subsequent purification using Ni^2+^-mediated affinity chromatography. Generally, WK6 cells were cultured in Terrific Broth (TB) medium supplemented with 0.1% (*w*/*v*) glucose, 100 μg/mL ampicillin, and 2 mM MgCl_2_. When the culture reached the exponential growth phase, 1 mM IPTG was added to the culture medium to induce the production of Nbs during overnight incubation at 28 °C. After collection of the cells by centrifugation, Nbs expressed in the periplasm were released by osmotic shock, and then purified by a two-step purification strategy. Firstly, immobilized metal affinity chromatography (IMAC) was performed to capture Nbs through His-tag with HisPur™ Ni-NTA Resin (Thermo Scientific, Carlsbad, CA, USA) and eluted by 500 mM imidazole. Then, the eluted fractions containing Nbs were further purified by size exclusion chromatography (SEC) on a Superdex™ 75 10/300 GL column (GE Healthcare, Waukesha, WI, USA). The purity and identity of obtained Nbs were confirmed by SDS-polyacrylamide gel electrophoresis (SDS-PAGE) and Western Blot. The concentration of Nbs was determined by measuring the absorbance at 280 nm using the theoretical extinction coefficient. Nbs were adjusted to a concentration of 1 mg/mL, aliquoted and stored at −80 °C until further use.

### 2.5. Binding Confirmation of Selected Nbs to SF

The targeting of the selected Nbs to SF in a native conformation was confirmed by immune dot blots. Briefly, 2 μL of SF was spotted on the nitrocellulose (NC) membrane. Then, the residual protein binding sites on the membrane were blocked with 3% skimmed milk. After washing with PBST, Nbs were incubated with the membrane and shaken to allow the potential recognition of the absorbed SF. Spots with PBS instead of antigen served as the blank control. The Nbs present on spots with native SF were visualized after sequential incubation with anti-His IgG and HRP conjugated anti-mouse IgGs, followed by color development in the developing solution (solution A: 6 mL methanol + 18 mg 4-chloro-1-Naphthol; solution B: 30 mL TPA solution (500 mL: 14.63 g NaCl, 1.4 g Trizma base, pH 7.5) + 18 μL H_2_O_2_).

### 2.6. Specificity and Cross-Reactivity of Nbs

The binding capacity and cross-reactivity of the selected Nbs was validated by ELISA. Briefly, SF or other irrelevant proteins including human albumin, trophoblast cell-surface antigen-2 (TROP-2), and epithelial cell adhesion molecule (EpCAM) at a concentration of 1 μg/mL were coated overnight at 4 °C in a microtiter plate. After sealing with 3% skim milk, Nbs at a concentration of 5 μg/mL were added to facilitate the binding to the coated antigen. After washing with PBST, mouse anti-His IgG and HRP-conjugated goat anti-mouse IgG were incubated successively for 1 h at RT. 3,3′,5,5′-Tetramethylbenzidine (TMB) substrate was used for color development, and the absorbance value at 450 nm was recorded by using a microplate reader (Tecan, Männedorf, Switzerland) after the termination of the color reaction with 2 M H_2_SO_4_ solution.

### 2.7. Characterization of Selected Nbs

#### 2.7.1. Binding Affinity

The apparent affinity of selected Nbs was estimated by saturated ELISA. Briefly, 100 μL of SF at a concentration of 1 μg/mL was coated in microtiter plates. After blocking the remaining protein binding sites with 3% skim milk, serially diluted Nbs were incubated with antigen for 1 h at RT. PBS was used as the blank instead of Nbs. Mouse anti-His tag IgG and HRP-conjugated goat anti-mouse IgG were used as the primary and secondary antibodies, respectively. TMB substrate was used for color development, and the absorbance value was determined with a microplate reader at 450 nm after the termination of the reaction with 2 M H_2_SO_4_. The apparent affinity was estimated as the molar concentration of the Nb yielded half of the maximum response.

#### 2.7.2. Thermal Stability

The thermal stability of Nbs was inferred by the melting temperature (Tm) as scored using thermofluor in CFX Connect™ Real-Time PCR Detection System (Bio-Rad, Hercules, CA, USA). In general, Nbs were ultrafiltered to a final concentration of 2.5 mg/mL. In a total reaction volume of 30 μL were mixed 15 μL of Nb, 5 μL of SYPRO^®^ Orange Protein Gel Stain (1/50 diluted, Sigma-Aldrich, St. Louis, MO, USA), and 10 μL of sterile PBS. The blank containing only PBS and Orange Protein Gel Stain was set up for the baseline control. The assessment was in triplicate for each Nb. The temperature was raised from 25 °C to 95 °C at a rate of 0.5 °C/min with the fluorescence signal recorded continuously. The obtained data was analyzed to determine the melting temperature (Tm) of Nbs after a non-linear fitting.

### 2.8. Development of Nb-Based Immunoassay

#### 2.8.1. Expression and Purification of Nbs with His-Tag Only

To develop the Nb-based heterologous sandwich ELISA, Nbs with only His-tag were used as the capturing antibodies in this study. Briefly, the gene fragments encoding the specific Nbs were subcloned into the pHEN6c plasmids to form recombinant vectors and then transformed into *E. coli* WK6 cells. The clones were confirmed by colony-PCR and sequencing with primers of M13F and M13R. The protocol for induction and expression of His-tagged Nbs was similar to that applied for the preparation of Nbs with HA- and His-tag. Also, the purification strategy, including IMAC and SEC was identical. The purity and identity of His-tagged Nbs were demonstrated by SDS-PAGE and Western Blot. The binding property of His-tagged Nbs against SF was verified by ELISA.

#### 2.8.2. Selection of Best Nb-Pair and Concentration Optimization for SF Detection

Based on the selected Nbs, a heterologous sandwich ELISA was designed to develop the immune detection of SF, using the His-tagged Nbs as capturing antibodies, and Nbs with HA- and His-tag as detecting antibodies. Firstly, the optimal Nb-pair was determined by performing a checkerboard ELISA. His-tagged Nbs at 10 μg/mL were coated in wells of a microtiter plate after 1 h incubation at RT. After blocking with 3% skim milk, 100 μL of SF (1 μg/mL) was supplemented and incubated for 1 h at RT. Then, 100 μL of Nbs with His- and HA-tag at the concentration of 5 μg/mL were incubated. Mouse anti-HA IgG and HRP-conjugated goat anti-mouse IgG were sequentially added. Then, the TMB substrate served for the color development. After the termination of the reaction with 2 M H_2_SO_4_, the optical density was recorded at 450 nm. The best performing Nb-pair was selected as the optimal Nb-pair. Thereafter, the concentration of the capturing and detecting Nbs were optimized by ELISA according to the steps described above. Different concentrations of capturing and detecting Nbs (1, 2, 3, 4, 6, and 7 μg/mL) were used. The Nb-based immunoassay was repeated at least twice.

#### 2.8.3. Development of Nb-Based Immunoassay

After confirmation of the best performing Nb-pair and the concentration optimization, serially diluted antigens (2500, 1000, 500, 300, 180, 100, 80, 50, 30, 10, 3, and 1 ng/mL) were applied to the developed sandwich ELISA for determination of a calibration curve and the linear standard using the antigen concentration and OD_450nm_ value as the abscissa and ordinate, respectively. The capturing and detecting antibodies were used at the optimal concentration as determined in the previous section. Generally, diluted antigens were added to wells with immobilized capturing Nbs and incubated for 1 h. Then, 5 washing steps with PBST were included to remove unbounded proteins, and detecting Nbs were added to stain the captured SF during 1 h incubation. Mouse anti-His tag IgG (1:5000) and goat anti-mouse IgGs (1:5000) were added sequentially to allow the staining in 1 h incubation. After washing, TMB substrate was added for color development during the incubation of 3 min. The absorbance at 450 nm was recorded by a microplate reader after stopping the reaction with 2 M H_2_SO_4_. The calibration equation was determined after non-linear fitting (four-parameter logistic curve, 4PL), and linear standard after linear regression. The cut-off value was confirmed by calculating the response from the blank groups (*n* = 10). The limit of detection (LOD) and limit of quantitation (LOQ) was defined as the protein concentration corresponding to the average cut-off value plus 3 or 10 times the standard deviations (SD), respectively.

#### 2.8.4. Analysis of Serum Sample

The applicability of the established ELISA was validated by simulating the detection of SF spiked in fetal bovine serum (FBS). A different concentration of SF was added into FBS and then centrifuged at 2500 rpm for ten minutes. The supernatant was collected as the sample for detection with the established ELISA. The recovery percentage and coefficient of variation (CV) were calculated according to the results to assess the precision and reproducibility of the sandwich ELISA.

### 2.9. Statistical Analysis

The data were analyzed by GraphPad Prism 7 (GraphPad Software Inc., San Diego, CA, USA) and the results were presented as mean ± SEM.

## 3. Results

### 3.1. Construction of an Immune Nb Library

**Figure 2:**
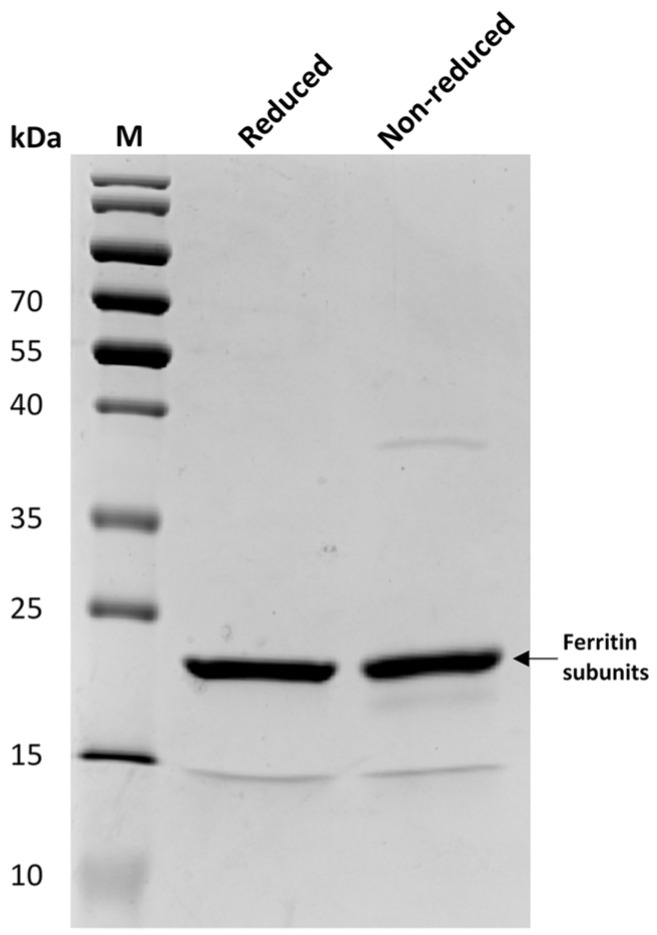
The antigen protein of SF was analyzed by SDS-PAGE. SDS-PAGE was performed under reduced (lane Reduced) and non-reduced (lane Non-reduced) conditions to indicate the size and distribution of SF subunits. The protein size standard (lane M) with its molecular mass (kDa) is shown on the left side.

The molecular size of the SF antigen was assessed firstly by SDS-PAGE (Figure 2) revealing a clear band with a size of around 20 kDa under either reducing (with β-mercaptoethanol) or non-reducing (without β-mercaptoethanol) conditions. This was anticipated to represent the bands of L and H subunits with sizes of around 20 kDa (theoretically 19 and 21 kDa) [36,37,38]. The band between 35–40 kDa visualized under non-reduced rather than reduced conditions was probably representing aggregates of L and H subunits of SF. In general, an acceptable purity of SF ensured that the major immune response would be directed against the envisaged target rather than against possible contaminants upon immunization.

The immunization was performed in a young and lively alpaca, and the peripheral blood lymphocytes were collected three days after the last boost to initiate the preparation of total RNA and following cDNA synthesis. The cDNA encoding VHHs were then amplified in a two-step nested PCR. After the first PCR fragments, encoding VH-CH1-CH2 and VHH-CH2 with a size of around 900 or 700 bp, respectively, were obtained (Figure S1A). The fragments of ~700 bp were extracted and served as the template in the second PCR to amplify VHH fragments with a size around 400 bp (Figure S1B). The VHH amplicons were ligated into pMECS phagemids and the resulted recombinant vectors were transformed into *E. coli* TG1 cells to construct the immune library with the determined size of 1.7 × 10^7^ colony forming units (CFU)/mL. The percentage of bacteria (randomly chosen) containing the phagemid with an insertion with the size of a VHH encoding gene was determined by colony PCR and agarose gel electrophoresis, which revealed a yield of 91% clones with an insert of the correct size (Figure S1C). Hence, an immune library against SF has been constructed with an adequate size and quantity of inserts.

### 3.2. Retrieval of SF-Specific Nbs

**Figure 3:**
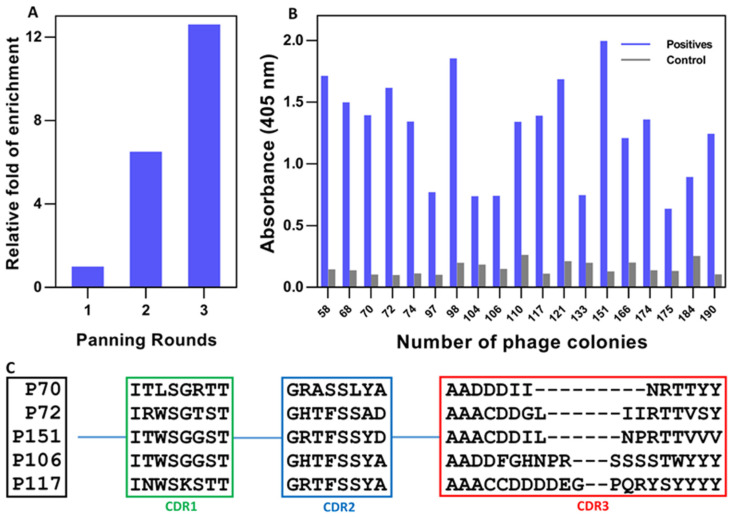
Selection of SF-specific Nbs after bio-panning and screening. (**A**) The relative enrichment of panning rounds. (**B**) The potential positive binders after the screening of specific Nbs by PE-ELISA. (**C**) The amino acid sequence of SF-specific Nbs after sequencing and family classification.

After constructing the Nb immune library, consecutive rounds of bio-panning using increasingly stringent washings were performed to enrich phages displaying SF-specific Nbs. The relative enrichment during three rounds of bio-panning was obvious (Figure 3A), which revealed the presence of increasing numbers of phages with target-specific Nbs during the consecutive rounds of panning.

To identify the ferritin-specific Nbs, 190 individual colonies (63 from the 1st round, 95 from the 2nd round, and 32 from the 3rd round of bio-panning) were cherry-picked and cultured for the preparation of periplasmic extracts containing Nbs with HA- and His-tag in order to investigate their potential recognition of the immobilized SF target in a PE-ELISA. The results revealed a total of 19 positive colonies with a response of at least twice that of the blank (without SF coated in the wells) (Figure 3B). These were subsequently classified, mainly according to their nucleotide sequence difference within the CDR3 loop, as Nbs from five different families and referred to as Nb70, Nb72, Nb106, Nb117, and Nb151 (Figure 3C).

### 3.3. Purification of Selected Nbs and Binding Analysis

**Figure 4:**
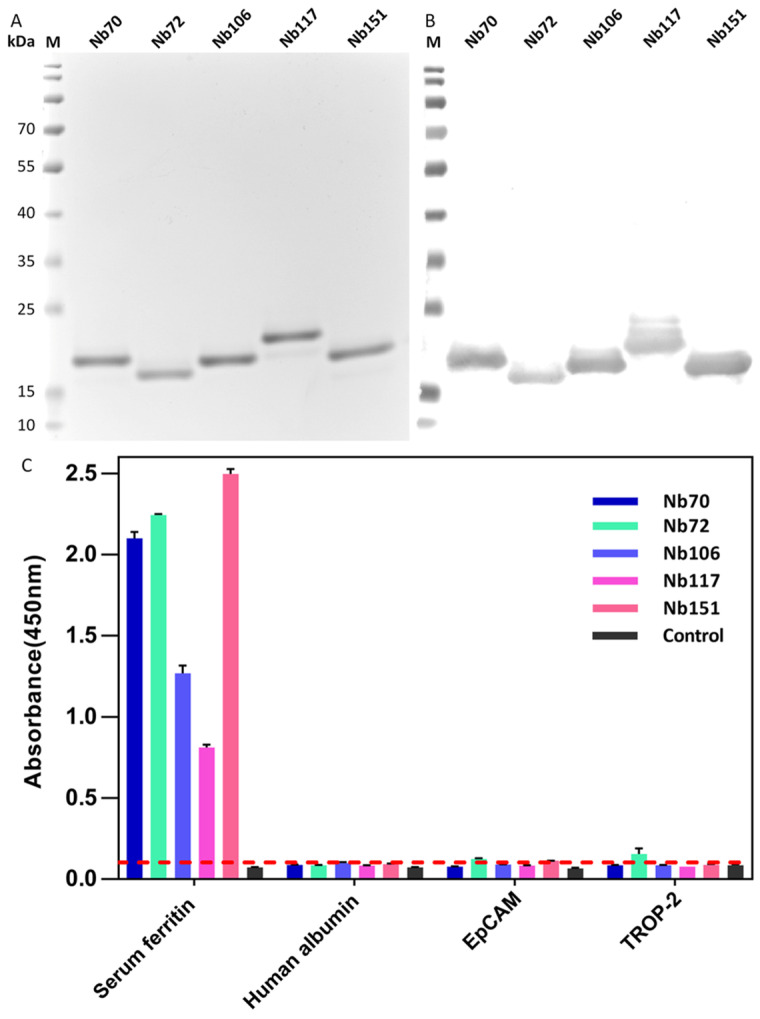
Preparation and confirmation of selected Nbs. (**A**) The purity and molecular size of HA- and His-tagged Nbs was verified by SDS-PAGE. (**B**) The identity of selected Nbs was analyzed by Western Blot. (**C**) Indirect ELISA was performed to detect binding specificity of selected Nbs and cross-reactivity with other proteins such as human albumin, EpCAM, and TROP-2. The cut-off value below 0.1 was considered the threshold for no cross-reaction. All data are expressed as mean ± SD (*n* = 3) and repeated at least 3 times.

The recombinant plasmids containing the genes encoding selected Nbs were extracted and transformed into *E. coli* WK6 cells for the expression of HA- and His-tagged monomeric Nbs. The periplasmic expression of Nbs was induced after supplementing IPTG to the bacterial culture, and a two-step purification strategy including IMAC and SEC was used to purify the selected Nbs. The high yield of selected Nbs ranged from 10–15 mg/L culture medium (Table S2). The purity and molecular size of Nbs was confirmed by SDS-PAGE to reveal a single band with a size of around 15 kDa, coinciding with the expected molecular mass of Nbs (Figure 4A). The identity of the purified Nb bands was verified by Western Blot to possess the His-tag (Figure 4B).

### 3.4. Target Specificity and Possible Cross-Reactivity

The binding specificity and cross-reactivity of selected Nbs were determined against SF, and other irrelevant proteins including human albumin, EpCAM, and TROP-2 in an ELISA. For the assessment with ELISA, a signal threshold below 0.1 was considered to lack cross-reactivity. As shown in Figure 4C, a significant response was raised against SF for all selected Nbs indicating the association with the cognate SF antigen, and no cross-reaction was observed with other irrelevant targets including human albumin, EpCAM, and TROP-2. In summary, it was demonstrated that the selected Nbs were characterized to have a high specificity to SF and no cross-reactivity was detected with irrelevant proteins.

### 3.5. Characterization of Selected Nbs

#### 3.5.1. Estimation of Affinity

The binding characteristic of selected Nbs was reflected by the apparent affinity estimation in a saturated ELISA. The fitting curves were drawn to determine the concentration of Nb yielding half of the maximum signal response, which corresponded roughly to the apparent affinity (Figure S2). As summarized in Table S2, the obtained apparent affinities were between 0.96–74.51 nM, with the relatively highest affinity observed for Nb72 and Nb70.

#### 3.5.2. Thermal Stability

The thermal stability of selected Nbs was deduced from their melting temperature as determined by the thermofluor assay. As shown in Figure S3, the resistance to the unfolding of all Nbs when exposed to elevated temperature was obvious and yielded Tm values ranging from 60 °C to 73 °C (Table S2). The highest stability was obtained for Nb72 and Nb106. The data demonstrated the conformity of the selected Nbs for the development of an immunoassay.

### 3.6. Development of Nb-Based Immunoassay

**Figure 5:**
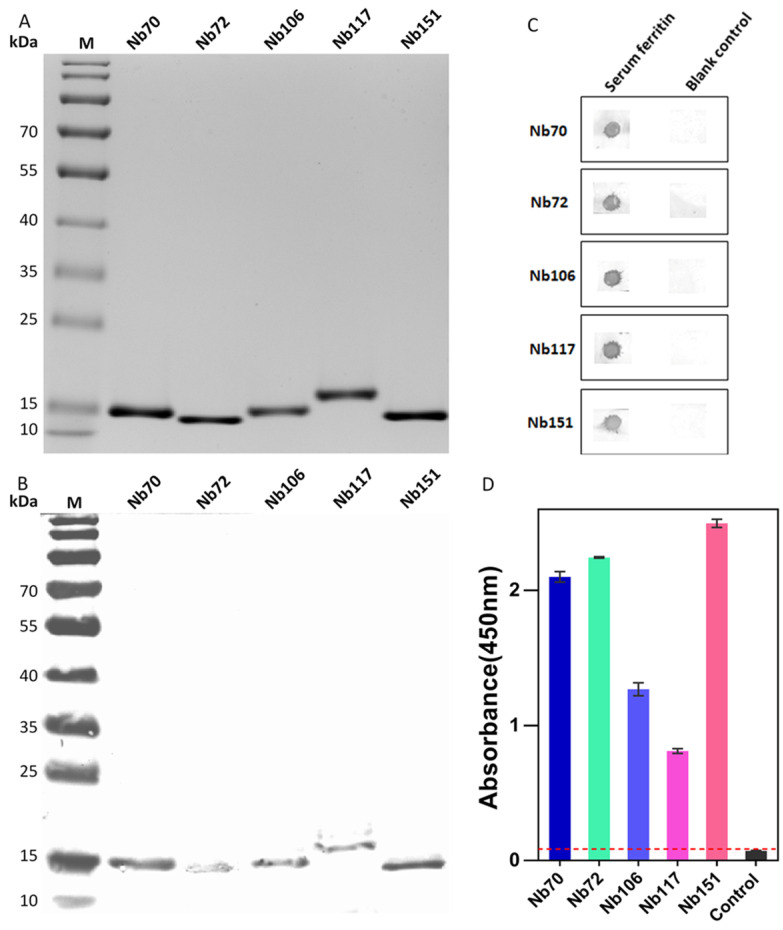
Preparation and characterization of selected Nbs fused with only His-tag. (**A**) The purity and molecular size of His-tagged Nbs was confirmed by SDS-PAGE. (**B**) The identity of His-tagged Nbs was analyzed by Western Blot after sequential staining with mouse anti-His tag IgG and goat anti-mouse IgGs. (**C**) The binding of selected Nbs to the native format of SF protein was verified by performing dot immune blotting, with the SF coated groups indicated as Serum ferritin and the control groups as the blank control. (**D**) Indirect ELISA was performed to detect the binding capacity of His-tagged Nbs. All data are expressed as mean ± SD (*n* = 3) and were repeated at least 3 times.

To develop a heterologous Nb-based sandwich ELISA for detecting SF, the best Nb-pair for capturing and detecting Nbs needs to be identified. In this study, Nbs with only His-tag or decorated with both tags (HA- and His-tag) were used as capturing or detecting Nbs, respectively. Thus, Nb with only His-tag needed to be prepared first. His-tagged Nbs were produced in the periplasm of *E. coli* WK6 after the subcloning of the VHH encoding genes into our pHEN6c plasmid. The same purification steps were applied for the preparation of Nbs with HA- and His-tag. Generally, a yield of approximately 10 mg/L culture medium was observed. The purity and identity of His-tagged Nbs were confirmed by SDS-PAGE and Western Blot, which revealed single bands corresponding to the size of Nbs (Figure 5A), and the presence of His-tag (Figure 5B). The binding of the selected Nbs to the native format of SF was assessed either in a dot blot assay or in an ELISA. The binding of the selected Nbs to the native conformation of SF protein was visualized on the NC membrane after the color development of the blot. The results demonstrated a clear association of the Nbs with the native antigen when compared with the blank controls devoid of signal (Figure 5C). For the ELISA assessment, a significant response was noted against SF for all selected Nbs, which indicated the recognition of the cognate SF antigen (Figure 5D).

**Figure 6:**
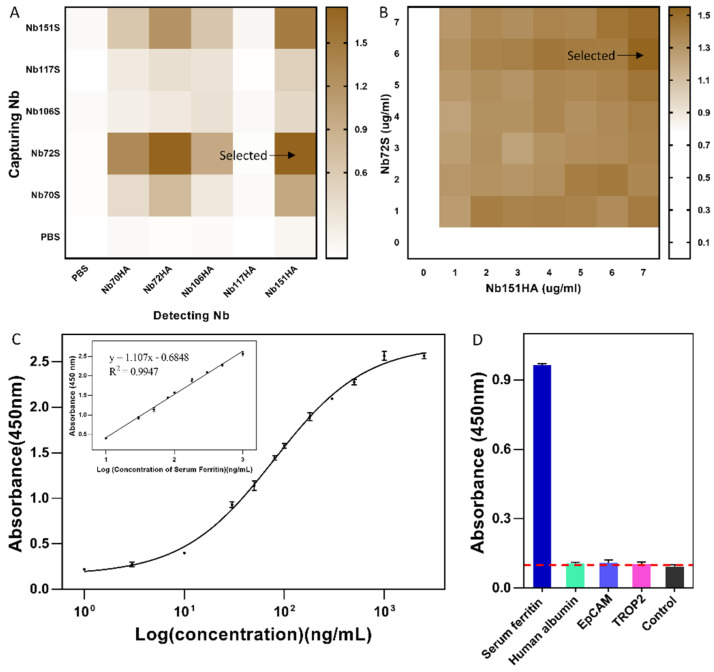
Establishment of an Nb-based sandwich ELISA method for SF detection. (**A**) Selection of the best performing Nb-pair to develop an immunoassay. Nb72S and Nb151HA were identified as capturing and detecting Nb, respectively. (**B**) Optimization of the concentration of capturing and detecting Nbs. In this instance, 6 μg/mL and 7 μg/mL were chosen for capturing Nb72S and detecting Nb151HA, respectively. (**C**) Calibration curve and the embedded linear standard curve of the developed sandwich ELISA. (**D**) Specificity and cross-reactivity of the developed sandwich ELISA. The specificity and cross-reactivity of the developed immunoassay were determined against SF, and irrelevant proteins of human albumin, EpCAM, and TROP2. The cut-off value below 0.1 was used as the threshold for the absence of cross-reactivity. All data are expressed as mean ± SD (*n* = 3) and were repeated at least 3 times.

After purification of capturing Nbs, the optimal pair of Nbs was determined by performing checkerboard ELISA. The Nb-pair with the best performance was identified by comparing the positive (P, sample with SF) and negative (N, sample without SF) signal. As shown in Figure 6A, the highest response was observed for Nb-pair Nb72 and Nb151, which were selected as the best pair of Nbs to develop an immunoassay.

Then, a similar approach was followed to determine the optimal concentration of capturing and detecting Nbs (Nb72 and Nb151), to ensure the highest detection sensitivity and lowest background. As shown in Figure 6B, the optimal concentration was determined as 6 μg/mL and 7 μg/mL for Nb72 and Nb151, respectively.

According to the optimal conditions determined in previous investigations, an Nb-based immunoassay was established. A calibration curve was determined by analyzing the absorbance values of SF at concentrations ranging from 1 to 2500 ng/mL. As shown in Figure 6C, the regression equation for the standard curve was generated: y = 1.107lgx − 0.6848 (R^2^ = 0.9947, *n* = 3). The best linear range of this detection method was obtained between 9.0–1.1 × 10^3^ ng/mL. The LOD and LOQ of the developed sandwich ELISA has been determined at 1.007 ng/mL and 2.109 ng/mL, respectively. In conclusion, a novel immunoassay based on selected Nbs was established to quantify SF. The cross-reactivity of the developed sandwich ELISA was further confirmed by evaluating the signal against SF and other irrelevant proteins (human albumin, EpCAM, and TROP-2). As shown in Figure 6D, the results demonstrated no cross-reactivity in the developed immunoassay. The results demonstrated the detection efficiency and applicability of this ELISA with specificity for SF.

### 3.7. Analysis of Serum Samples

The applicability of the developed immunoassay was further investigated by detecting samples of FBS spiked with different concentrations of human SF. The spiked samples were centrifuged to remove excess particles, and the supernatant was collected for detecting SF with the established assay. As shown in Table 1, the reliability of the established immunoassay was evaluated by determining SF recovery that ranged from 104% to 112% with a CV of less than 10%, which demonstrated the successful application of the developed immunoassay for SF detection in spiked samples.

## 4. Discussion

SF is the protein tightly connected with iron storage and homeostasis, with a clear utility as a clinical tool to assess body iron levels. As an important clinical biomarker, SF content measurement can not only reveal the iron status, but also reflects a morbid state such as iron-deficiency anemia. Iron-deficiency anemia is an important risk affecting adolescents, women, and the elderly worldwide [39]. Further investigation has increasingly verified the prognostic practice of SF variation with a multitude of other disorders like neurodegenerative and malignant diseases [12,40,41]. The SF level is also markedly elevated during chronic and acute inflammation to correlate with an increased level of c-reactive protein (CRP) and alpha 1-acid glycoprotein (AGP) [42,43]. In patients influenced by inflammatory or infectious diseases, the quantification of SF level can assist the identification of inflammation-related anemia from iron-deficiency anemia by simultaneously considering other indicators of iron deficiency such as soluble transferrin receptor (STfR) [44]. Generally, the measurement and assessment of SF content is of great importance as a prognostic marker to reflect the iron status, and potential disorders such as inflammation, infection, or malignancy.

Various methods have been developed for the detection of SF levels, including RIA and ELISA. Barnett et al. used I^125^ to label ferritin so that the labeled ferritin and unlabeled ferritin competed for the binding to antibodies. This competitive detection method resulted in a detection range between 2.5–350 ng/mL [45]. Bleicher et al. reported a sandwich ELISA using polyclonal antibodies for the quantification of ferritin with a detection range between 3.2–232 ng/mL [46]. Garg et al. reported microfluidics-based nanoparticle-enhanced electrochemical detection by using an integrated electrochemically active screen-printed electrode (SPE) to monitor SF with a detection range between 7.81–500 ng/mL [23]. Priyadarshini et al. conjugated ferritin antibodies to gold@carbon dot nanoconjugates, with which they realized an immunodetection in the range of 1–120 ng/mL [47]. All of the detecting approaches described above utilized conventional antibodies in a monoclonal or polyclonal format to trace the level of SF. However, the drawbacks of conventional antibodies include their high production cost, being labor-intensive, and the difficulties of manipulation. In addition, a too low affinity of the antibodies resulted in a restricted sensitivity and applicability of the developed immunoassay. The unique properties of Nbs facilitate their excellent performance in detecting disease-related biomarkers for clinical practice [48,49]. Thus, it is worthy to explore the preparation of specific Nbs against the clinical SF biomarker, and their potential as immunodetecting reagents for the clinical surveillance of SF.

In this study, SF-specific Nbs were successfully retrieved after immunization and selection procedures. The robust characteristics of selected Nbs were verified by determining the excellent thermal stability and high affinity against SF. In order to verify the applicability of the selected Nbs as diagnostic reagents, a heterologous Nb-pair-based sandwich ELISA was proposed to be established. The parameters demonstrated the sensitivity and broad detection range of the developed immunoassay. The applicability of the detection approach was also confirmed by quantifying SF spiked in serum samples. More importantly, it is promising to conjugate the selected Nbs with more sensitive detection techniques like fluorescence- or chemiluminescence-based detection methods to further enhance the sensitivity and applicability.

In conclusion, SF-specific Nbs have been successfully generated, and their potential as diagnostic reagents has been investigated by developing an immunoassay in order to provide a method for clinical application. More importantly, this study confirmed the successful application of Nbs to detect disease-related biomarkers and potentially extend the research and application of Nbs for clinical diagnosis and therapy.

## Figures and Tables

**Table 1:** Recoveries of serum ferritin in serum samples.

Sample	Spiked Concentration	Detected Concentration	Recovery ^b^	CV ^c^
	ng/mL	ng/mL	%	%
FBS ^a^	0	ND ^d^	-	-
	10	11.13 ± 1.03	111.25%	3.64%
	100	104.20 ± 2.26	104.20%	2.14%
	500	548.75 ± 20.53	109.75%	8.70%

^a^ Fetal bovine serum. ^b^ Each assay was repeated three times, and the result for recovery was the average of three replicates. ^c^ CV was the ratio of the standard deviation to the mean. ^d^ Non-detectable.

## Supplementary Materials

The following supporting information can be downloaded at: https://www.mdpi.com/article/10.3390/biom12081080/s1, Table S1. Primers used in the study; Table S2. Properties of selected Nbs; Figure S1. Construction of the immune Nb library; Figure S2. Apparent bind affinity of selected Nbs; Figure S3. Thermal stability of selected Nbs.

## References

[1] Wang W., Knovich M.A., Coffman L.G., Torti F.M., Torti S.V. (2010). Serum ferritin: Past, present and future. Biochim. Et Biophys. Acta-Gen. Subj..

[2] Jacobs A., Worwood M. (1975). Ferritin in serum. Clinical and biochemical implications. N. Engl. J. Med..

[3] Plays M., Muller S., Rodriguez R. (2021). Chemistry and biology of ferritin. Metallomics.

[4] Sammarco M.C., Ditch S., Banerjee A., Grabczyk E. (2008). Ferritin L and H subunits are differentially regulated on a post-transcriptional level. J. Biol. Chem..

[5] Koperdanova M., Cullis J.O. (2015). Interpreting raised serum ferritin levels. BMJ.

[6] Knovich M.A., Storey J.A., Coffman L.G., Torti S.V., Torti F.M. (2009). Ferritin for the clinician. Blood Rev..

[7] Lopez A., Cacoub P., Macdougall I.C., Peyrin-Biroulet L. (2016). Iron deficiency anaemia. Lancet.

[8] Daru J., Allotey J., Pena-Rosas J.P., Khan K.S. (2017). Serum ferritin thresholds for the diagnosis of iron deficiency in pregnancy: A systematic review. Transfus. Med..

[9] Georgieff M.K. (2020). Iron deficiency in pregnancy. Am. J. Obstet. Gynecol..

[10] Abioye A.I., McDonald E.A., Park S., Ripp K., Bennett B., Wu H.W., Pond-Tor S., Sagliba M.J., Amoylen A.J., Baltazar P.I. (2019). Maternal anemia type during pregnancy is associated with anemia risk among offspring during infancy. Pediatric Res..

[11] Cullis J.O., Fitzsimons E.J., Griffiths W.J., Tsochatzis E., Thomas D.W., British Society for Haematology (2018). Investigation and management of a raised serum ferritin. Br. J. Haematol..

[12] Fan K., Gao L., Yan X. (2013). Human ferritin for tumor detection and therapy. Wiley Interdiscip. Rev. Nanomed. Nanobiotechnol..

[13] Hu Z.W., Chen L., Ma R.Q., Wei F.Q., Wen Y.H., Zeng X.L., Sun W., Wen W.P. (2021). Comprehensive analysis of ferritin subunits expression and positive correlations with tumor-associated macrophages and T regulatory cells infiltration in most solid tumors. Aging (Albany N. Y.).

[14] Zandman-Goddard G., Shoenfeld Y. (2007). Ferritin in autoimmune diseases. Autoimmun. Rev..

[15] Batchelor E.K., Kapitsinou P., Pergola P.E., Kovesdy C.P., Jalal D.I. (2020). Iron Deficiency in Chronic Kidney Disease: Updates on Pathophysiology, Diagnosis, and Treatment. J. Am. Soc. Nephrol..

[16] Akpinar H., Cetiner M., Keshav S., Ormeci N., Toruner M. (2017). Diagnosis and treatment of iron deficiency anemia in patients with inflammatory bowel disease and gastrointestinal bleeding: Iron deficiency anemia working group consensus report. Turk. J. Gastroenterol..

[17] Means R.T. (2020). Iron Deficiency and Iron Deficiency Anemia: Implications and Impact in Pregnancy, Fetal Development, and Early Childhood Parameters. Nutrients.

[18] Byg K.E., Milman N., Hansen S., Agger A.O. (2000). Serum Ferritin is a Reliable, Non-invasive Test for Iron Status in Pregnancy: Comparison of Ferritin with Other Iron Status Markers in a Longitudinal Study on Healthy Pregnant Women; Erythropoiesis. Hematology.

[19] Crispin P., Stephens B., McArthur E., Sethna F. (2019). First trimester ferritin screening for pre-delivery anaemia as a patient blood management strategy. Transfus. Apher. Sci..

[20] Palmer W.C., Zaver H.B., Ghoz H.M. (2020). How I Approach Patients with Elevated Serum Ferritin. Am. J. Gastroenterol..

[21] Matysiak-Brynda E., Wagner B., Bystrzejewski M., Grudzinski I.P., Nowicka A.M. (2018). The importance of antibody orientation in the electrochemical detection of ferritin. Biosens. Bioelectron..

[22] Devarakonda S., Singh R., Bhardwaj J., Jang J. (2017). Cost-Effective and Handmade Paper-Based Immunosensing Device for Electrochemical Detection of Influenza Virus. Sensors.

[23] Garg M., Christensen M.G., Iles A., Sharma A.L., Singh S., Pamme N. (2020). Microfluidic-Based Electrochemical Immunosensing of Ferritin. Biosensors.

[24] Song T.-T., Wang W., Meng L.-L., Liu Y., Jia X.-B., Mao X. (2017). Electrochemical detection of human ferritin based on gold nanorod reporter probe and cotton thread immunoassay device. Chin. Chem. Lett..

[25] Tu Z., Huang X., Fu J., Hu N., Zheng W., Li Y., Zhang Y. (2020). Landscape of variable domain of heavy-chain-only antibody repertoire from alpaca. Immunology.

[26] Muyldermans S. (2021). A guide to: Generation and design of nanobodies. FEBS J..

[27] Oliveira S., Heukers R., Sornkom J., Kok R.J., van Bergen En Henegouwen P.M. (2013). Targeting tumors with nanobodies for cancer imaging and therapy. J. Control. Release.

[28] Garaicoechea L., Aguilar A., Parra G.I., Bok M., Sosnovtsev S.V., Canziani G., Green K.Y., Bok K., Parreno V. (2015). Llama nanoantibodies with therapeutic potential against human norovirus diarrhea. PLoS ONE.

[29] Salvador J.P., Vilaplana L., Marco M.P. (2019). Nanobody: Outstanding features for diagnostic and therapeutic applications. Anal. Bioanal. Chem..

[30] Haffke M., Fehlmann D., Rummel G., Boivineau J., Duckely M., Gommermann N., Cotesta S., Sirockin F., Freuler F., Littlewood-Evans A. (2019). Structural basis of species-selective antagonist binding to the succinate receptor. Nature.

[31] Jovcevska I., Muyldermans S. (2020). The Therapeutic Potential of Nanobodies. Biodrugs.

[32] Krah S., Schroter C., Zielonka S., Empting M., Valldorf B., Kolmar H. (2016). Single-domain antibodies for biomedical applications. Immunopharmacol. Immunotoxicol..

[33] Vincke C., Gutiérrez C., Wernery U., Devoogdt N., Hassanzadeh-Ghassabeh G., Muyldermans S. (2012). Generation of single domain antibody fragments derived from camelids and generation of manifold constructs. Methods Mol. Biol..

[34] Hu Y., Romao E., Vincke C., Brys L., Elkrim Y., Vandevenne M., Liu C., Muyldermans S. (2021). Intrabody Targeting HIF-1alpha Mediates Transcriptional Downregulation of Target Genes Related to Solid Tumors. Int. J. Mol. Sci..

[35] Pardon E., Laeremans T., Triest S., Rasmussen S.G., Wohlkonig A., Ruf A., Muyldermans S., Hol W.G., Kobilka B.K., Steyaert J. (2014). A general protocol for the generation of Nanobodies for structural biology. Nat. Protoc..

[36] Koorts A.M., Viljoen M. (2007). Ferritin and ferritin isoforms I: Structure-function relationships, synthesis, degradation and secretion. Arch. Physiol. Biochem..

[37] Song X., Zheng Y., Liu Y., Meng H., Yu R., Zhang C. (2022). Production of recombinant human hybrid ferritin with heavy chain and light chain in Escherichia coli and its characterization. Curr. Pharm. Biotechnol..

[38] Harrison P.M., Arosio P. (1996). The ferritins: Molecular properties, iron storage function and cellular regulation. Biochim. Et Biophys. Acta.

[39] Cappellini M.D., Musallam K.M., Taher A.T. (2020). Iron deficiency anaemia revisited. J. Intern. Med..

[40] Alkhateeb A.A., Connor J.R. (2013). The significance of ferritin in cancer: Anti-oxidation, inflammation and tumorigenesis. Biochim. Et Biophys. Acta.

[41] Zhang N., Yu X., Xie J., Xu H. (2021). New Insights into the Role of Ferritin in Iron Homeostasis and Neurodegenerative Diseases. Mol. Neurobiol..

[42] Ashktorab H., Pizuorno A., Aduli F., Laiyemo A.O., Oskrochi G., Brim H. (2021). Elevated Liver Enzymes, Ferritin, C-reactive Protein, D-dimer, and Age Are Predictive Markers of Outcomes Among African American and Hispanic Patients with Coronavirus Disease 2019. Gastroenterology.

[43] Ayoya M.A., Spiekermann-Brouwer G.M., Stoltzfus R.J., Nemeth E., Habicht J.P., Ganz T., Rawat R., Traore A.K., Garza C. (2010). Alpha 1-acid glycoprotein, hepcidin, C-reactive protein, and serum ferritin are correlated in anemic schoolchildren with Schistosoma haematobium. Am. J. Clin. Nutr..

[44] Braga F., Infusino I., Dolci A., Panteghini M. (2014). Soluble transferrin receptor in complicated anemia. Clin. Chim. Acta.

[45] Barnett M.D., Gordon Y.B., Amess J.A., Mollin D.L. (1978). Measurement of ferritin in serum by radioimmunoassay. J. Clin. Pathol..

[46] Bleicher A.V., Unger H.W., Rogerson S.J., Aitken E.H. (2018). A sandwich enzyme-linked immunosorbent assay for the quantitation of human plasma ferritin. MethodsX.

[47] Priyadarshini E., Rawat K., Bohidar H.B., Rajamani P. (2019). Dual-probe (colorimetric and fluorometric) detection of ferritin using antibody-modified gold@carbon dot nanoconjugates. Microchim. Acta.

[48] Bastos-Soares E.A., Sousa R.M.O., Gomez A.F., Alfonso J., Kayano A.M., Zanchi F.B., Funes-Huacca M.E., Stabeli R.G., Soares A.M., Pereira S.S. (2020). Single domain antibodies in the development of immunosensors for diagnostics. Int. J. Biol. Macromol..

[49] Yang E.Y., Shah K. (2020). Nanobodies: Next Generation of Cancer Diagnostics and Therapeutics. Front. Oncol..

