# Supplementary material for: Identification of Serum Ferritin-Specific Nanobodies and Development towards a Diagnostic Immunoassay
Source: Biomolecules. 2022 Aug 5;12(8):1080. doi: 10.3390/biom12081080 (PMC9406126; doi:10.3390/biom12081080)
Supplement: Supplementary file 1 [file biomolecules-12-01080-s001.zip › biomolecules-1827041-supplementary.pdf]

Supplementary Information

Identification of serum ferritin-specific nanobodies and development towards a diagnostic immunoassay

Yaozhong Hu[1, #], Jing Lin[1, #], Yi Wang[1], Sihao Wu[1], Jing Wu[1], Huan Lv[1], Xuemeng Ji[1], Serge Muyldermans[2], Yan Zhang[1, *], Shuo Wang[1, *]

1. Research Institute of Public Health, School of Medicine, Nankai University, Tianjin 300071, China.
2. Lab of Cellular and Molecular Immunology, Vrije Universiteit Brussel, 1050 Brussels, Belgium.

[#] These authors (Y. Hu and J. Lin) contribute equally to this work.

[*] Corresponding authors,

S. Wang: wangshuo@nankai.edu.cn

Y. Zhang: yzhang@nankai.edu.cn

Tel.: +86 22 85358445

Table S1. Primers used in this study

Primers	Sequence
CALL001	5’-GTC CTG GCTGCT CTT CTA CAA GG-3’
CALL002	5’-GGT ACG TGC TGT TGA ACT GTT CC-3’
PMCF	5’-CTA GTG CGG CCG CTG AGG AGA CGG TGA CCT GGG T-3’
A6E	5’-GAT GTG CAG CTG CAG GAG TCT GGR GGA GG-3’
MP57	5’-CCA CAG ACA GCC CTC ATA G-3’
GIII	5’-TTA TGC TTC CGG CTC GTA TG-3’
M13F	5’-CGCCAGGGTTTTCCCAGTCACGAC-3’
M13R	5’-TCACACAGGAAACAGCTATGAC-3’

Table S2 Properties of selected Nbs

Nbs	Mw/ kDa	pI -	Yield mg/L	Tm [a] ℃	K_D [a] nM
**Nb70**	15.02	8.66	12.44	60.97±0.20	2.10±0.60
**Nb72**	15.08	6.39	14.20	65.62±0.31	0.96±0.16
**Nb106**	15.65	7.96	8.50	72.75±0.12	27.15±6.40
**Nb117**	16.23	6.43	13.50	61.77±0.26	74.51±20.65
**Nb151**	15.12	9.07	15.20	60.59±0.48	6.18±1.17

[a] Results are represented as mean ± SD (n = 3). Repeated at least 3 times for every test.

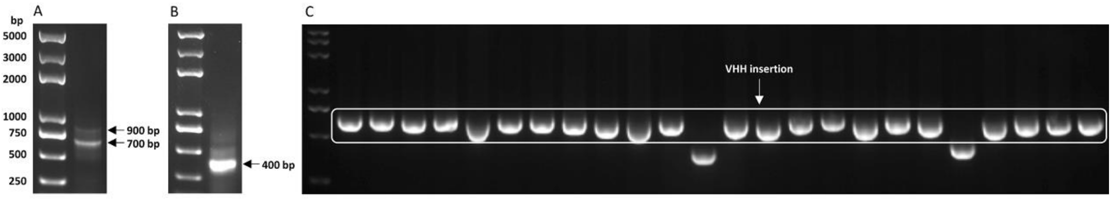

Figure S1. Construction of the immune Nb library. A Nb gene fragments were amplified by a first PCR. Bands of 700 bp were fragments encoding VHH-CH2, and bands of 900 bp represents the encoding fragments of VHH-CH1-CH2. B Nb gene fragments were amplified by a second PCR with the size of around 400 bp. C The correct insertion of VHH fragments was indicated as VHH insertion.

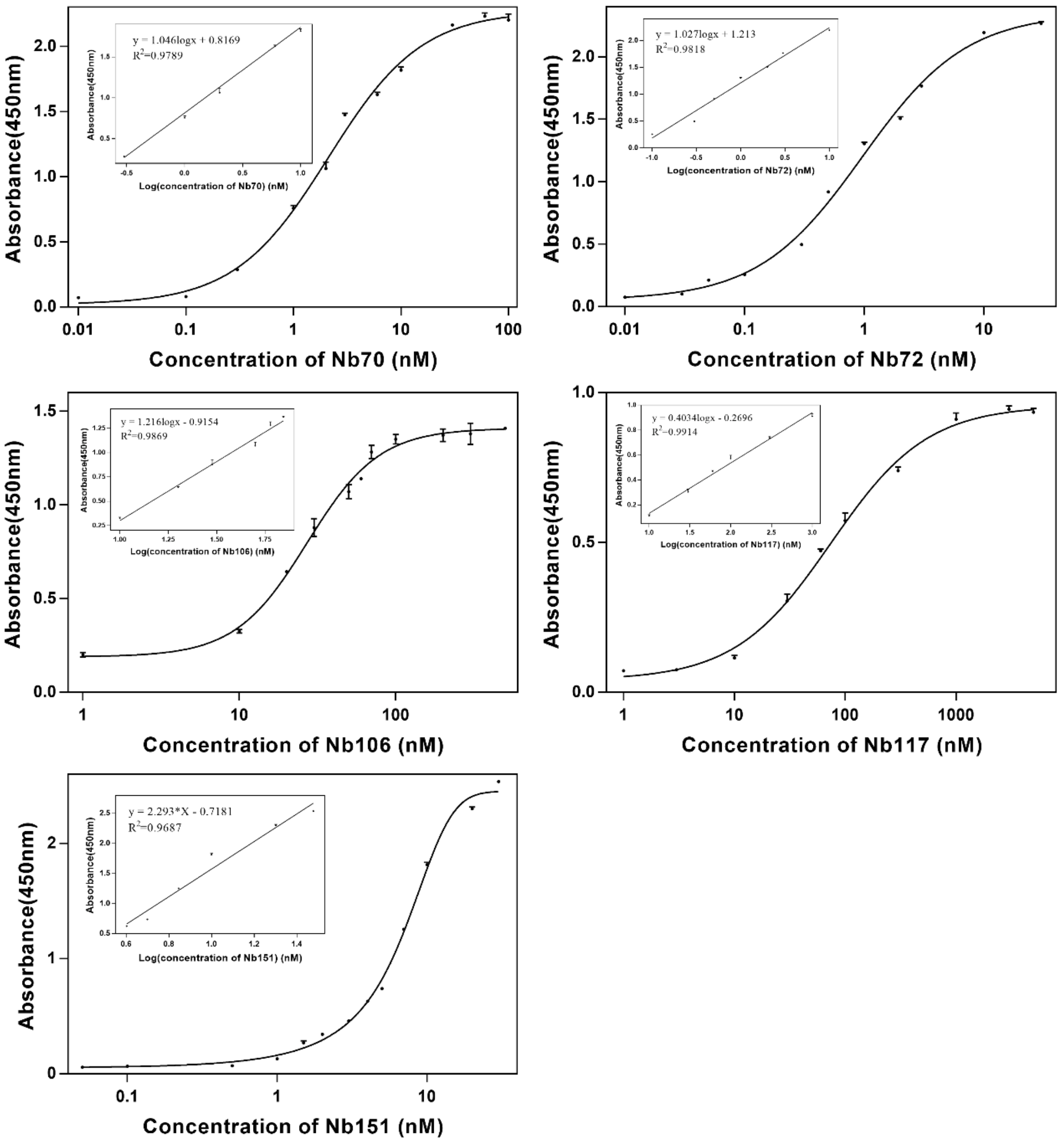

Figure S2. Apparent bind affinity of selected Nbs. Data was indicated as mean ± SD (n = 3), and repeated at least 3 times.

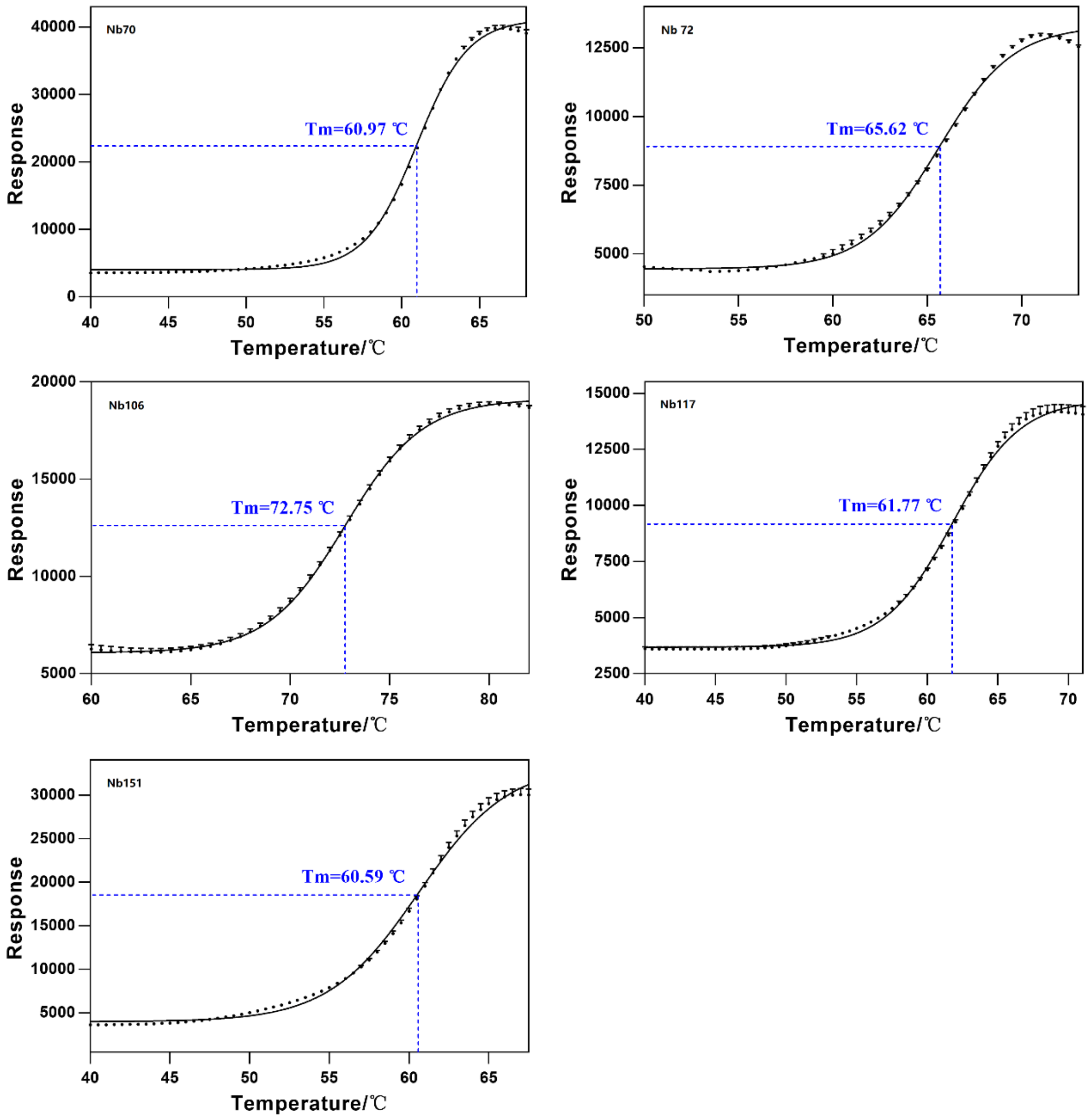

Figure S3. Thermal stability of selected Nbs. The thermal stability of specific Nbs was reflected by the melting temperature after determining the melting curves of selected Nbs. Data was indicated as mean ± SD (n = 3), and repeated at least 3 times.
